# *In-Operando* X-Ray Imaging for Sobering Examination of Aqueous Zinc Metal Batteries

**DOI:** 10.1007/s40820-025-01911-0

**Published:** 2025-12-01

**Authors:** Yuhang Dai, Hongzhen He, Mengzheng Ouyang, Jianuo Chen, Jie Lin, Haobo Dong, Guanjie He

**Affiliations:** 1 Christopher Ingold Laboratory, Department of Chemistry, University College London, London, WC1H 0AJ UK; 2 Present Address: Department of Engineering Science, University of Oxford, Oxford, OX1 3PJ UK; 3 Electrochemical Innovation Lab, Department of Chemical Engineering, University College London, London, WC1E 7JE UK; 4 Present Address: Department of Earth Science and Engineering, Imperial College London, London, SW7 2AZ UK; 5 School of Mechanical and Aerospace Engineering, Queen’s University Belfast, Belfast, BT9 5AH UK; 6 School of Future Technology, South China University of Technology, Guangzhou, 510641 People’s Republic of China

**Keywords:** Aqueous Zn metal batteries, X-ray imaging, *In situ* characterization, Degradation mechanism

## Abstract

Aqueous zinc metal batteries (AZMBs) face significant challenges in achieving reversibility and cycling stability, primarily due to hydrogen evolution reactions (HER) and zinc dendrite growth. In this study, by employing carefully designed cells that approximate the structural characteristics of practical batteries, we revisit this widely held view through *in-operando* X-ray radiography to examine zinc dendrite formation and HER under near-practical operating conditions. While conventional understanding emphasizes the severity of these processes, our findings suggest that zinc dendrites and HER are noticeably less pronounced in dense, real-operation configurations compared to modified cells, possibly due to a more uniform electric field and the suppression of triple-phase boundaries. This study indicates that other components, such as degradation at the cathode current collector interface and configuration mismatches within the full cell, may also represent important barriers to the practical application of AZMBs, particularly during the early stages of electrodeposition.

**Graphical Abstract:**

**Supplementary Information:**

The online version contains supplementary material available at 10.1007/s40820-025-01911-0.

## Highlights

- *In-operando* X-ray imaging reveals distinct behaviors between real-service-inspired and modified aqueous zinc metal batteries (AZMBs).
- Densely packed setups show suppressed Zn dendrites and hydrogen evolution compared to modified cells.
- Findings suggest cathode degradation may also critically impact AZMB failure, beyond the anode limitations

**Supplementary Information:**

The online version contains supplementary material available at 10.1007/s40820-025-01911-0.

## Graphical abstract

**Figure:**
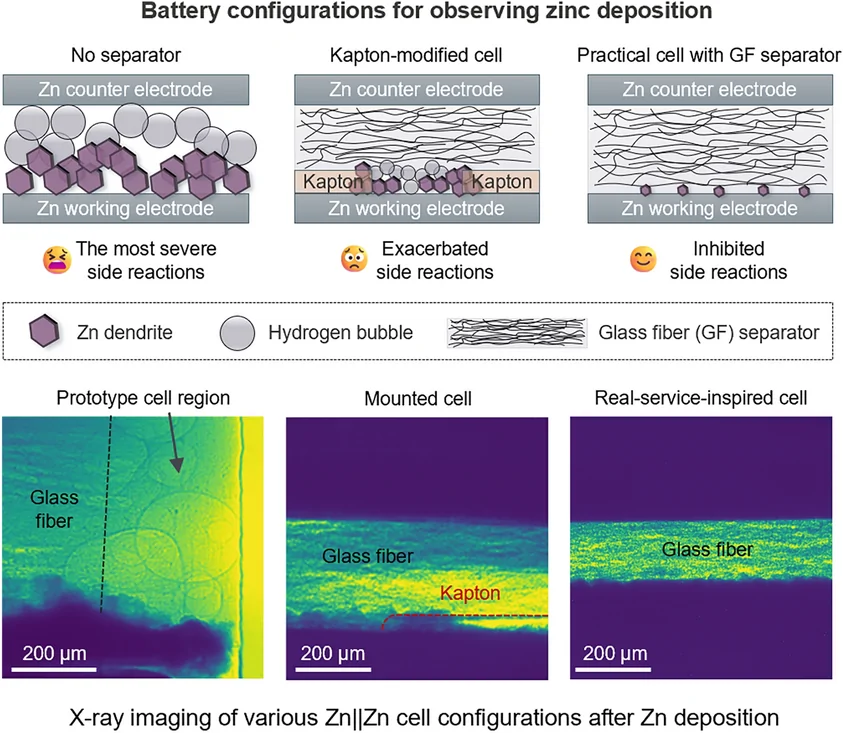

## Introduction

Zinc (Zn) metal anodes have garnered significant attention due to their high theoretical capacity of 820 mAh g^−1^, cost-effectiveness, sustainability, and stability in aqueous solutions. When paired with water-based electrolytes, aqueous zinc metal batteries (AZMBs) offer intrinsic safety and high ionic conductivity, making them promising candidates for fast-charging applications. Despite these merits, practical deployment of AZMBs has remained limited, primarily due to their inadequate cycle life that is often associated with hydrogen evolution reactions (HER) and Zn dendrite growth [1].

Although Zn dendrites typically grow as hexagonal sheets and are generally considered less detrimental compared to spike-like Li metal dendrites, under certain conditions they can still contribute to localized short circuits and performance degradation [2, 3]. As a result, dendrite growth is often regarded as a secondary issue relative to HER, with the latter causing significant cell swelling and detachment of electrodes from separators, posing a major concern [4]. Conventional *in-situ* and *ex-situ* investigations using methods like optical microscopy (OM), atomic force microscopy (AFM), and electron microscopies, as well as other techniques (summarized in Table 1), have shown that bubbles generated by HER present more visible challenges than Zn dendrites [5–7]. These bubbles undergo nucleation, growth, and diffusion, with their behaviors differing between densely packed real batteries and modified characterization cells that introduce voids to enhance observation.

**Table 1:** Characterization tools for observing Zn metal deposition

Test condition	Method	Reference
*Ex-situ*	Scanning electron microscope	[13]
	Scanning transmission electron microscope	[14]
	Confocal laser scanning microscope	[15]
	Electron backscatter diffraction	[16]
	Time-of-flight secondary ion mass spectrometry	[17]
	Ellipsometry	[18]
*In-situ*	Atomic force microscopy	[19]
	Optical microscopy	[20]
	Transmission electron microscopy	[21]
*In-operando*	Fast X-ray radiography	This work

High-energy X-ray imaging is a non-destructive and advanced approach for probing internal cell structures by fully penetrating the entire cell [8]. This technique enables real-time observations in gently adjusted cell configurations, more closely reflecting real operational conditions without introducing artificial voids [9]. Compared to previous optical and electron microscopy-based techniques, high-energy X-ray radiography offers a greater penetration depth while preserving the integrity of the original cell architecture. Through optimizing testing parameters specifically for zinc-based aqueous batteries, our work advances *in-operando* characterization for AZMBs. In this work, synchrotron X-ray radiography was used to detect actual electrochemical processes and structural evolution within AZMBs. Owing to its high resolution and short collection time, it enables *in-operando* and *in-situ* observations of early-stage internal processes. Our results indicate that, under the specific testing conditions, Zn dendrite growth and HER appear notably mitigated in AZMBs with densely packed electrodes and separators, suggesting that the severity of these issues may have been overestimated under conventional evaluation setups. This mitigation is possibly linked to a more uniform electric field and the inhibition of continuous triple-phase (electrode–electrolyte–hydrogen bubble) boundary formation in densely configured cells compared to modified ones. These findings motivate future investigations into alternative degradation factors affecting the cycling stability of practical AZMB formats such as coin, pouch and prismatic cells. Potential areas for further study include cathode dissolution at low current densities [10], edge discharge phenomena [11], and the contact integrity between current collectors and active materials [12].

## Experimental Section

### Materials and Cell Assembly

Copper foil (thickness of 60 μm) and zinc foil (thickness of 100 μm), each with 99.9% purity, were used as positive and negative electrodes, respectively, in the synchrotron X-ray experiment. For the operando tests shown in Fig. S4, the Zn foils were pre-cycled in a separate Zn||Zn symmetric Swagelok cell at a current density of 1 mA cm^−2^ with a capacity of 1 mAh cm^−2^ for 5 cycles prior to reassembly. The 2 M ZnSO_4_ electrolyte used in all cells was prepared by dissolving 0.1 mol ZnSO_4_·7H_2_O and diluting to a final volume of 50 mL with deionized water, followed by vigorous magnetic stirring for 20 min. The electrodes were precisely cut into 12-mm-diameter discs and affixed to titanium collectors to provide strong, corrosion-resistant support. A Whatman® glass microfiber membrane (grade GF/D, 2.7 μm pore size) was selected, while a 20 μm thick Kepstan® PEKK (polyether–ketone–ketone) film served as a spacer between the electrodes and membrane. The PEKK layer facilitated unobstructed observation of zinc dendrite formation and HER. PFA Swagelok-type cells (1/2″) were assembled in a vertical configuration: copper foil, PEKK, GF/D membrane, PEKK, and zinc foil. This setup was specifically optimized for operando imaging of zinc-based aqueous batteries, bridging the gap between experimental models and practical cell configurations. By minimizing artificial voids and maintaining structural stability, this design closely resembles the electrochemical environment of pouch, coin and prismatic cells.

### Electrochemical Testing

Discharge tests were conducted at a current density of 15 mA cm^−2^ at room temperature with a Gamry Instruments Interface 1000E potentiostat. Open-circuit voltage (OCV) was measured until a steady fluctuation within ± 5 mV was achieved. Cells were discharged asymmetrically to induce zinc dendrite deposition on the copper foil, while HER occurred at the bottom electrode, simulating operational stresses.

### X-Ray Radiography

X-ray radiography was conducted at beamline I13-2 of the Diamond Light Source. A 30 keV monochromatic X-ray beam, focused to a 6.5 μm × 6.5 μm spot size, was used for cell imaging. The cell was mounted vertically on the sample stage, aligned parallel to the beam for detailed *in-situ* tomographic observations with a 0.5-s exposure time. The high frequency of bubble and dendrite formation necessitated selecting a stable cross section to ensure reliable operando 2D data collection.

### Imaging Equipment Details

A pco.edge 5.5 camera equipped with a 10× objective lens provided a total magnification of 20×. The effective pixel size was 0.325 μm, with a field of view of 0.83 mm × 0.7 mm. This configuration allowed for high-resolution imaging, essential for capturing subtle changes at the electrode–electrolyte interface during operation.

## Results and Discussion

### Visualization of Zn Deposition in Different Configurations

**Fig. 1:**
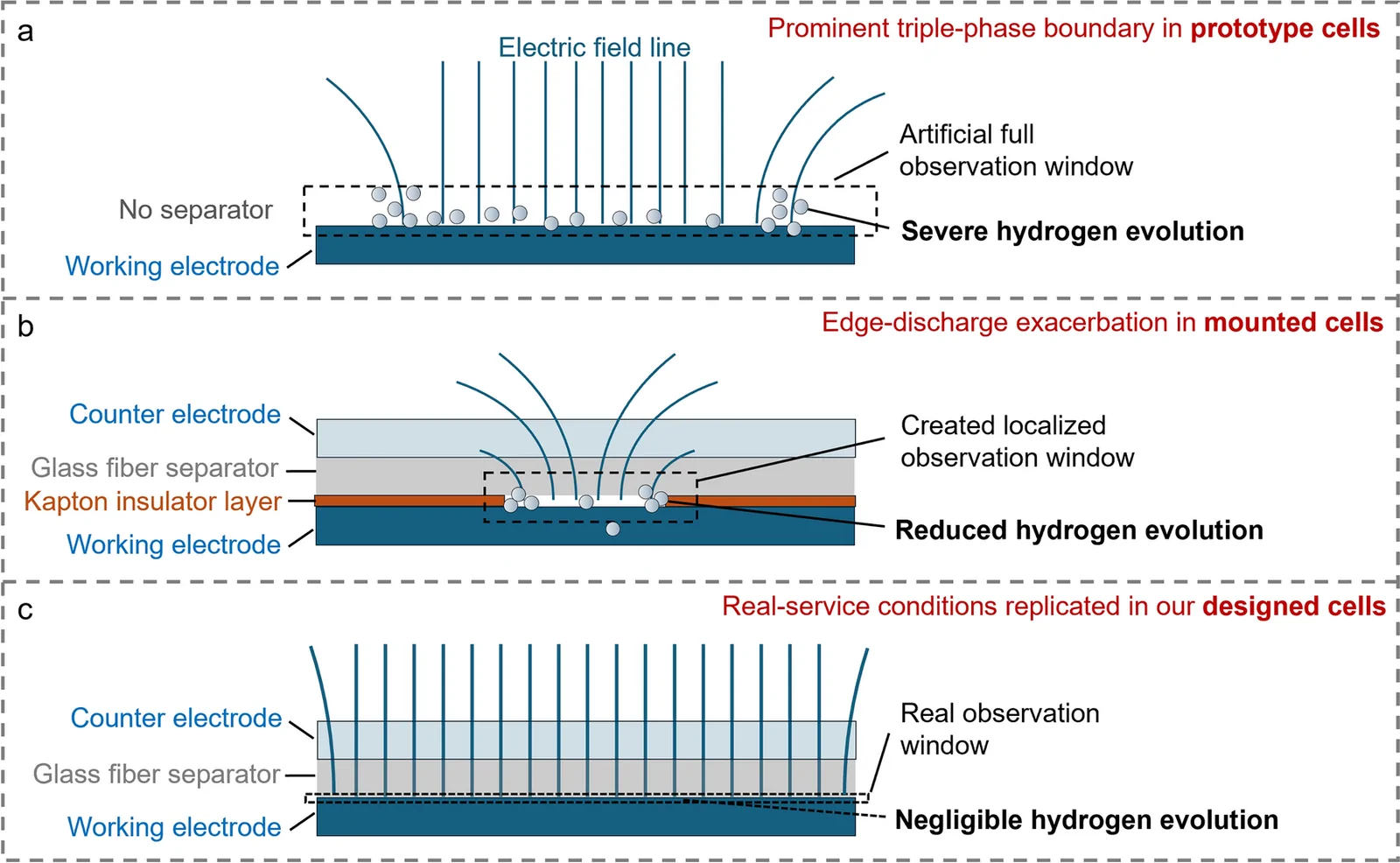
Schematic of configurations for observing Zn deposition. **a** Prototype cell. **b** Mounted cell. **c** Our designed cell reflecting real-service conditions

The prototype cell (Fig. 1a) was constructed without a separator on the metal working electrode, leading to a pronounced solid–liquid–gas triple-phase boundary and non-uniform electric fields, particularly at the edges. The mounted cell (Fig. 1b) introduced a localized observation window using a Kapton layer as an insulating barrier, enabling more detailed analysis of electrode dynamics within a confined region of interest (ROI). The Kapton layer, with a dielectric constant distinct from other cell components, spatially confines the observation region and introduces minor electric field perturbations near the observation window edges. However, this design amplified edge discharge effects compared to the prototype cell. In contrast, our designed real-service-inspired cell (Fig. 1c) more closely emulates practical configurations, such as coin or pouch cells, by employing a dense structure without artificial voids. This setup helps maintain uniform electric fields across the majority of the electrode and provides valuable insights into the actual behavior of Zn anodes under operational conditions. The electric field remained relatively uniform in regions near the cell center, while some distortion at the electrode edge. Due to the near-surface region in parallel with the electrolyte-immersed membrane (glass fiber separator), the electric field was more uniform and parallel compared to the prototype cell.

**Fig. 2:**
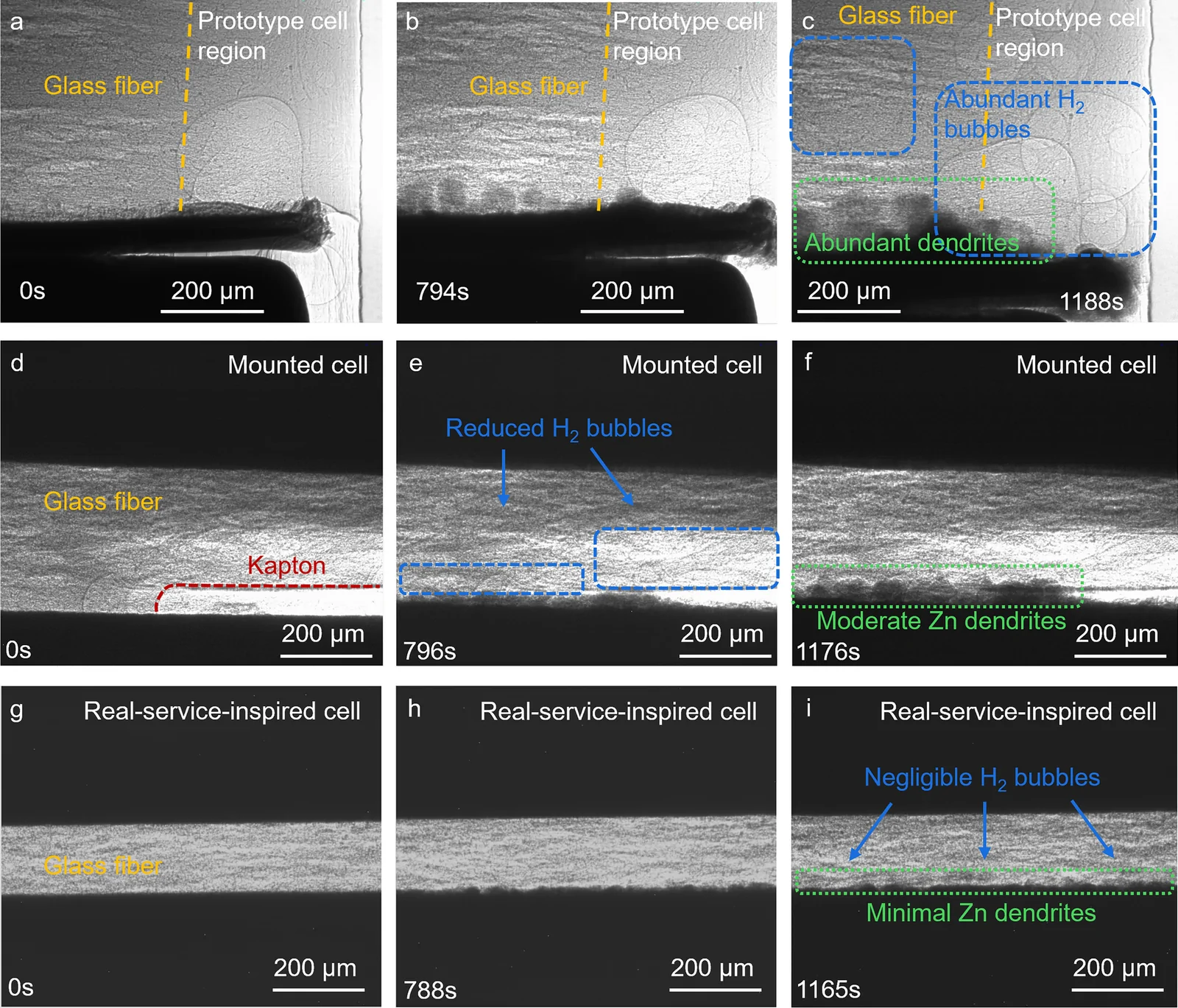
*Operando* X-ray radiography images of electrode–electrolyte interface within Swagelok cells. The galvanostatic discharge current density is 15 mA cm^−2^. **a-c** Regions with and without (Prototype cell) glass fiber separators indicated by the orange dash lines. **d-f** Mounted cell. **g-i** Designed real-service cell

These cell configurations were tested using *in-operando* X-ray radiography. The most pronounced reactions were observed in prototype cells (Fig. 2a-c), where an obvious triple-phase boundary was present, resembling the results from previous *in-situ* studies of side reactions in AZMBs [1]. Compared to prototype cells, the mounted cell configuration exhibited reduced HER but demonstrated a more severe edge discharge phenomenon, leading to Zn dendrite accumulation at the glass fiber-Kapton-Zn anode interface (Fig. 2d-f). Supporting trends were also observed via *in-situ* optical microscopy (Fig. S1), where the prototype cell exhibited the most severe HER, followed by the mounted cell, while the real-service-inspired cell showed the mildest HER. Moreover in the X-ray observation, the Zn dendrites in mounted cells exhibited a gradual outward slope from the interface (Fig. S2). However, in the above two encapsuled configurations, HER was not as pronounced as in previous studies. In contrast, Zn dendrites and HER were substantially alleviated in the real-service-inspired cells (Fig. 2g-i), suggesting that anodic side reactions may not be the main cause of degradation in full aqueous cells using Zn metal anodes, such as Zn||MnO_2_ cells and Zn||V_2_O_5_ cells. Consistent with these observations, prototype and mounted cells exhibited failure earlier than the real-service-inspired cells due to the intensified Zn dendrite formation and HER. Interestingly, after extended cycling, mounted cells failed even earlier than the prototype cells, likely due to more pronounced dendrite formation and HER at the electrode edges (Fig. S3). Figure S4 illustrates that dendrite growth is promoted on pre-corroded Zn foils, shedding light on the conditions that exacerbate side reactions where side reactions on its surface accelerate exponentially once the Zn metal corrodes. As a result, prototype and mounted cells may overestimate Zn dendrite formation and HER under typical testing conditions.

### Electrochemical Modeling to Understand Different Zn Deposition Behaviors

**Fig. 3:**
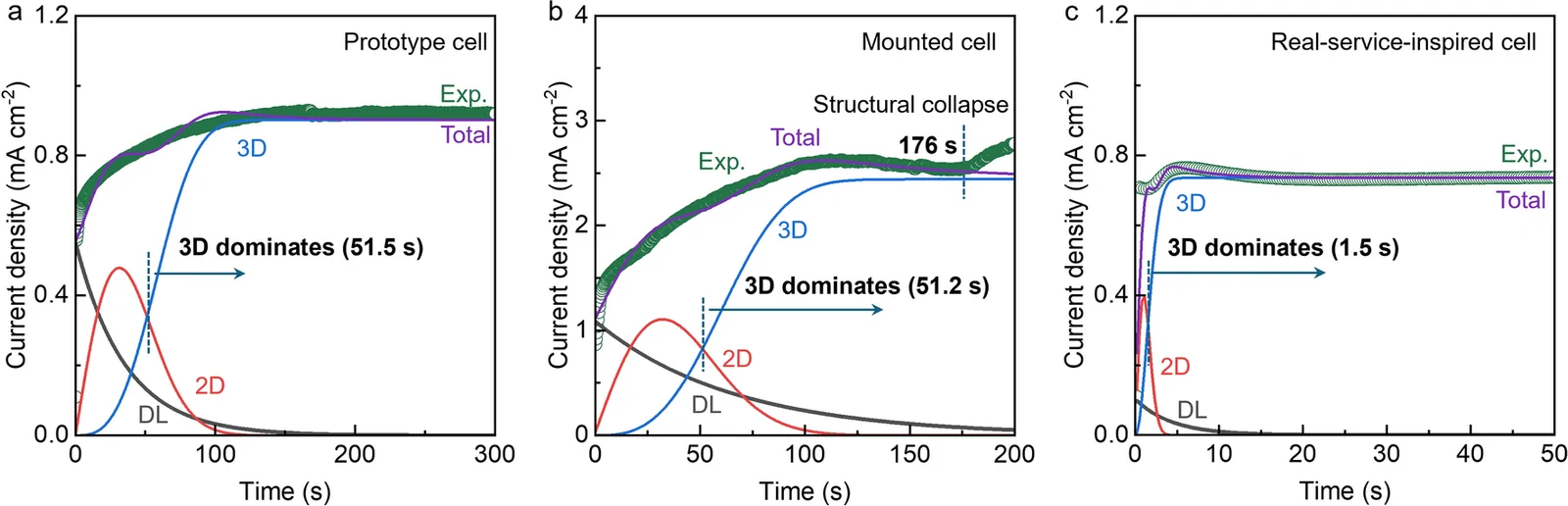
Chronoamperograms (CAs) at an overpotential of -150 mV. **a** Prototype cell. **b** Mounted cell. **c** Designed real-service cell. The DL, 2D, and 3D represent double-layer, instantaneous two-dimensional, and progressive three-dimensional currents. The long-term CA curves are shown in Fig. S5. Total and Exp. represent the simulated total current and the experimentally measured current, respectively

**Fig. 4:**
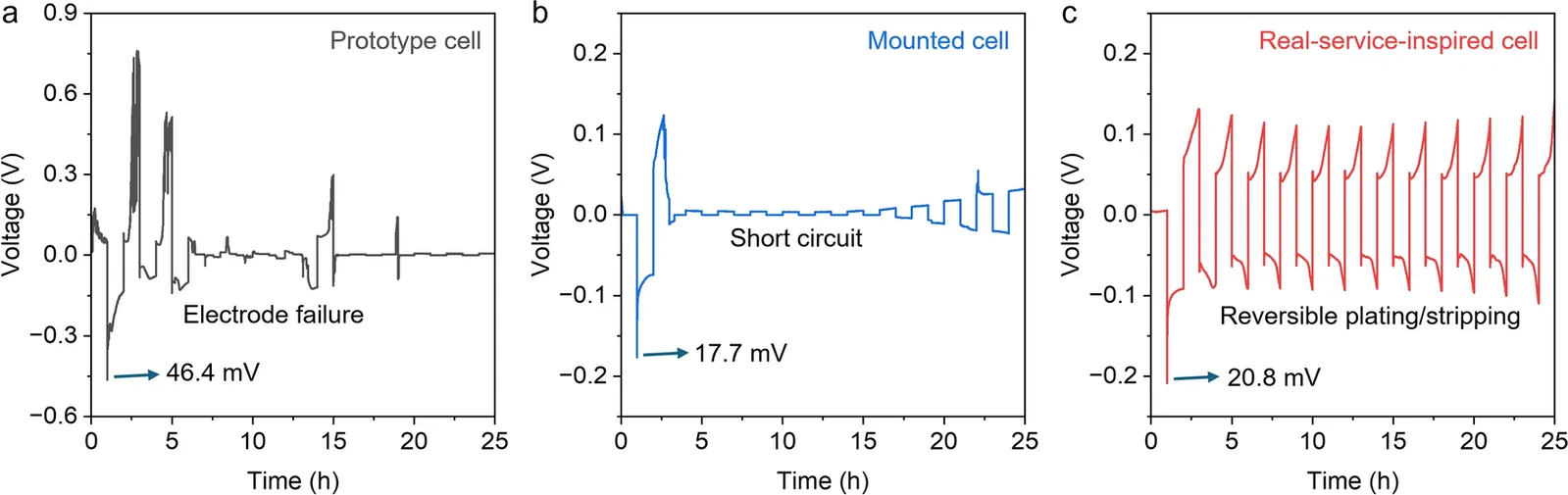
Galvanostatic cycling curves of Zn||Zn symmetric cells at 1 mA cm^−2^ with a fixed capacity of 1 mAh cm^−2^. **a** Prototype cell. **b** Mounted cell. **c** Designed real-service cell

Chronoamperometry (CA) measurements based on symmetrical Zn||Zn cells provided further insights into the dynamic Zn deposition process in different configurations (Fig. 3a-c). The current response was decomposed into double layer (DL), instantaneous two-dimensional growth (2D), and progressive three-dimensional growth (3D) components, described by the following equations:

1 $${I}_{DL}={I}_{0}\cdot {e}^{\frac{-t}{{\tau }_{DL}}}$$

2 $${I}_{2D}=a\cdot t\cdot {e}^{-b\cdot {t}^{2}}$$

3 $${I}_{3D}=c\cdot (1-{e}^{-d\cdot {t}^{3}})$$

where *a*, *b*, *c*, *d*, *I*_0_, and *τ*_DL_ correspond to physicochemical parameters (see Supplementary Note) [22]. For prototype and mounted cells, the transition from 2D to 3D diffusion dominance begins at 51.5 and 51.2 s, respectively, while significant current fluctuation starts at 176 s in mounted cells, corresponding to the structural collapse of the Zn working electrode. This gradual progression indicated slow and uneven Zn growth on the anodes, leading to an enhanced irregular deposition and the dendrite formation. In contrast, the real-service-inspired cell exhibited a rapid transition from 2D to 3D diffusion dominance within only 1.5 s, highlighting more uniform nucleation and growth of Zn. Consequently, these cells presented different behaviors in the galvanostatic charge–discharge cycling tests. Notably, the prototype and mounted cells exhibited stable cycling for less than 5 h (Fig. 4a, b), primarily due to severe Zn dendrite formation and HER issues observed in Fig. 2a-f. In contrast, the real-service-inspired cell maintained reversible deposition/stripping for over 25 h (Fig. 4c), consistent with the moderate side reactions shown in Fig. 2g-i. The lowest nucleation potential in mounted cells (17.7 mV) compared to prototype cells (46.4 mV) and real-service-inspired cells (20.8 mV) may be due to the narrow reaction window, which likely in turn exacerbated inhomogeneous Zn deposition.

### Postmortem and Electrode Replacement Analysis

To clarify the respective roles of anode and cathode degradation under practical full-cell conditions, we performed electrode replacement and postmortem analyses on Zn||MnO_2_ coin cells. As shown in Fig. S6, replacing the cycled cathode with a fresh one restored both capacity and capacity evolution trend, remaining consistent with pre-replacement performance. In contrast, replacing the Zn anode reversed the previously increasing capacity trend, leading to continuous fading. Morphological and structural characterizations (Figs. S7 and S8) further revealed more severe by-product accumulation and structural changes on the cathode side than on the anode side during cycling. These results suggest that cathode degradation may play a more significant role than anode degradation in limiting cell performance under the tested conditions.

## Conclusions

In summary, this study employed *in-operando* X-ray imaging technique coupled with carefully designed cell configuration to investigate the early stage behavior of Zn dendrites and HER in AZMBs. The results suggest that Zn dendrites and HER may be less severe in real-service-inspired cells compared to previous reports. This difference is likely associated with a more uniform electric field and the suppression of continuous triple-phase boundary formation in densely packed cell configurations. The high resolution and non-invasive nature of X-ray imaging enabled real-time visualization of the electrode–electrolyte interface during cell operation. These findings suggest that, under certain conditions reflecting practical conditions, limitations to AZMB cycling stability may arise more prominently from issues such as cathode material dissolution, edge discharge, and the integrity of contact among different cell components, rather than from Zn dendrites or HER alone. This insight may guide future research efforts toward addressing these factors to improve the performance of AZMBs in real-world scenarios.

## Supplementary Information

Below is the link to the electronic supplementary material.

Supplementary file1 (DOCX 28050 KB)
