# Supplementary material for: In-Operando X-Ray Imaging for Sobering Examination of Aqueous Zinc Metal Batteries
Source: Nanomicro Lett. 2025 Dec 1;18:85. doi: 10.1007/s40820-025-01911-0 (PMC12665639; doi:10.1007/s40820-025-01911-0)
Supplement: Supplementary file 1 — Supplementary file1 (DOCX 28050 KB) [file 40820_2025_1911_MOESM1_ESM.docx]

Supporting Information for

***In-Operando* X-Ray Imaging for Sobering Examination of Aqueous Zinc-Metal Batteries**

Yuhang Dai^1, 2, #^, Hongzhen He^3, 4, #^, Mengzheng Ouyang^4, #^, Jianuo Chen^3^, Jie Lin^5^, Haobo Dong^1, 6,^ * and Guanjie He^1,^ *

^1^ Christopher Ingold Laboratory, Department of Chemistry, University College London, London, WC1H 0AJ, UK

^2^ Department of Engineering Science, University of Oxford, Oxford, OX1 3PJ, UK

^3^ Electrochemical Innovation Lab, Department of Chemical Engineering, University College London, London, WC1E 7JE, UK

^4^ Department of Earth Science and Engineering, Imperial College London, London, SW7 2AZ, UK

^5^ School of Mechanical and Aerospace Engineering, Queen’s University Belfast, BT9 5AH, UK

^6^ School of Future Technology, South China University of Technology, Guangzhou 510641, People’s Republic of China

^#^Yuhang Dai, Hongzhen He, and Mengzheng Ouyang have contributed equally to this work.

*Corresponding author. E-mail: [dhbhubble@scut.edu.cn](mailto:dhbhubble@scut.edu.cn) (Haobo Dong); [g.he@ucl.ac.uk](mailto:g.he@ucl.ac.uk) (Guanjie He)

**Supplementary Figures and Table**


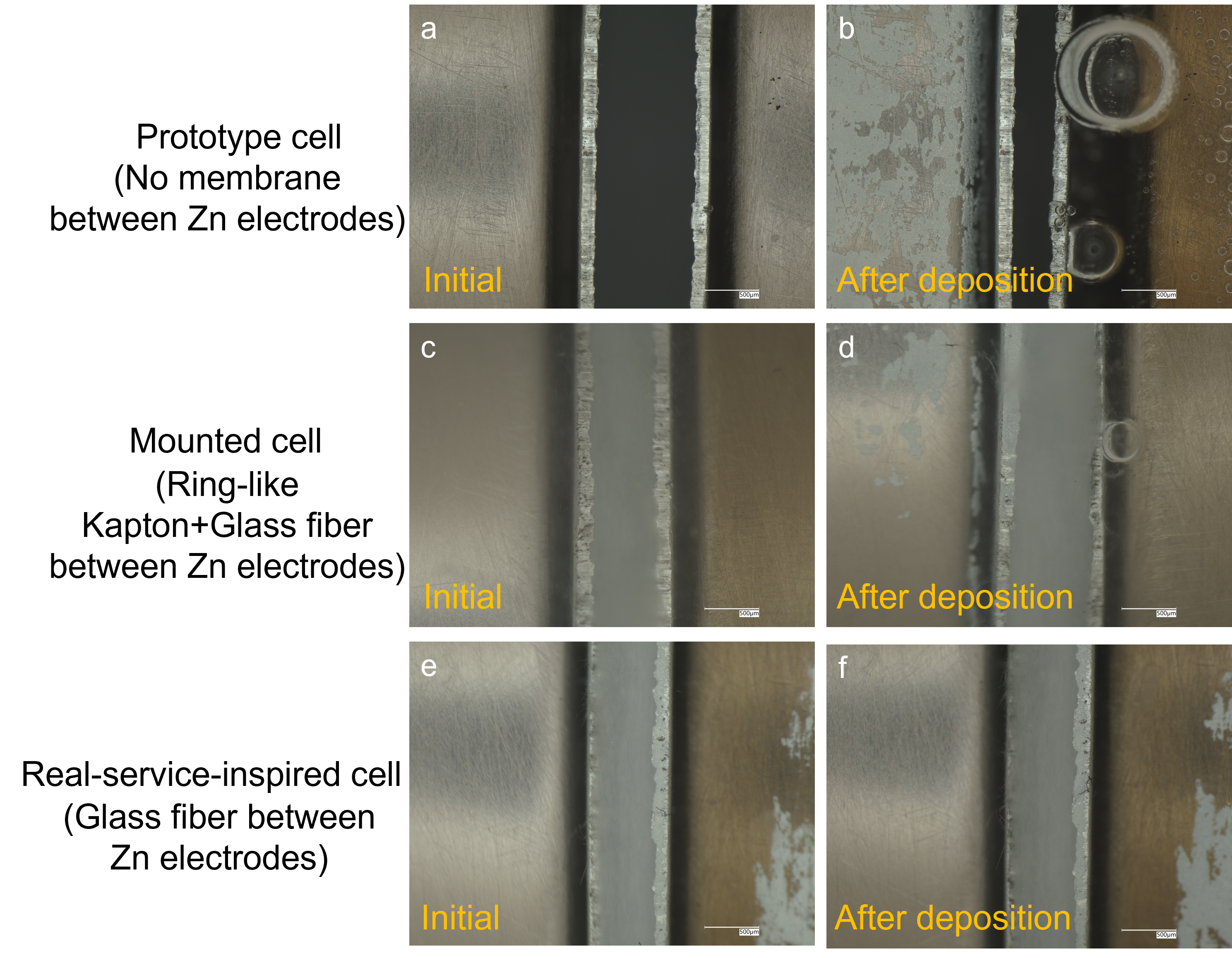


**Fig. S1** *In-situ* optical microscopy snapshots of Zn||Zn symmetric cells, showing the initial state and the morphology after 1,500 s of Zn deposition at 15 mA cm^-2^. (**a, b**) Prototype cell. (**c, d**) Mounted cell. (**e, f**) Real-service-inspired cell


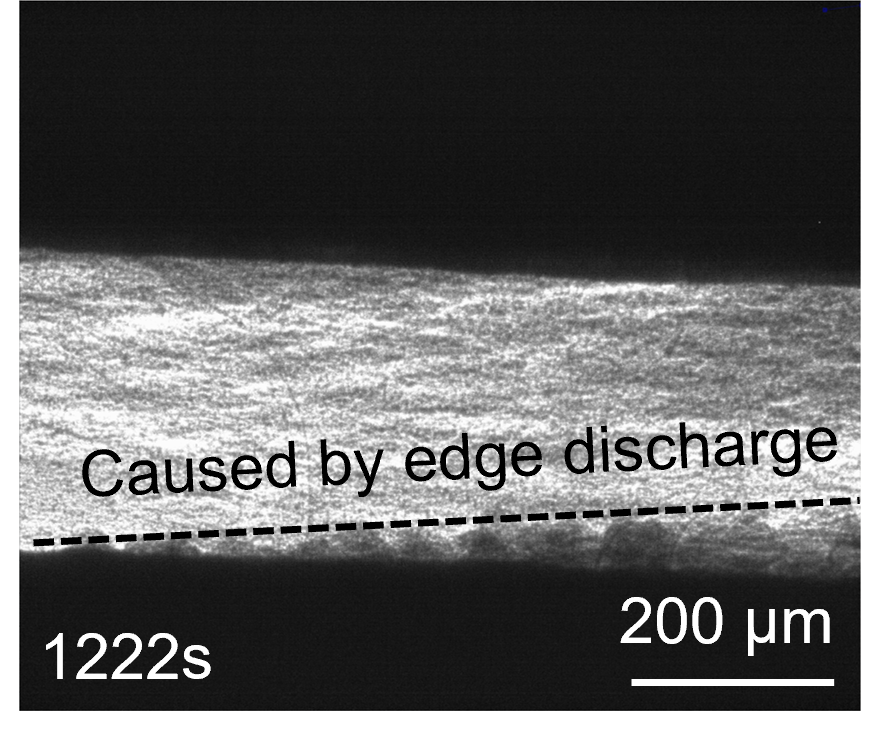


**Fig. S2** Snapshot recorded during operando X-ray imaging of the mounted cell


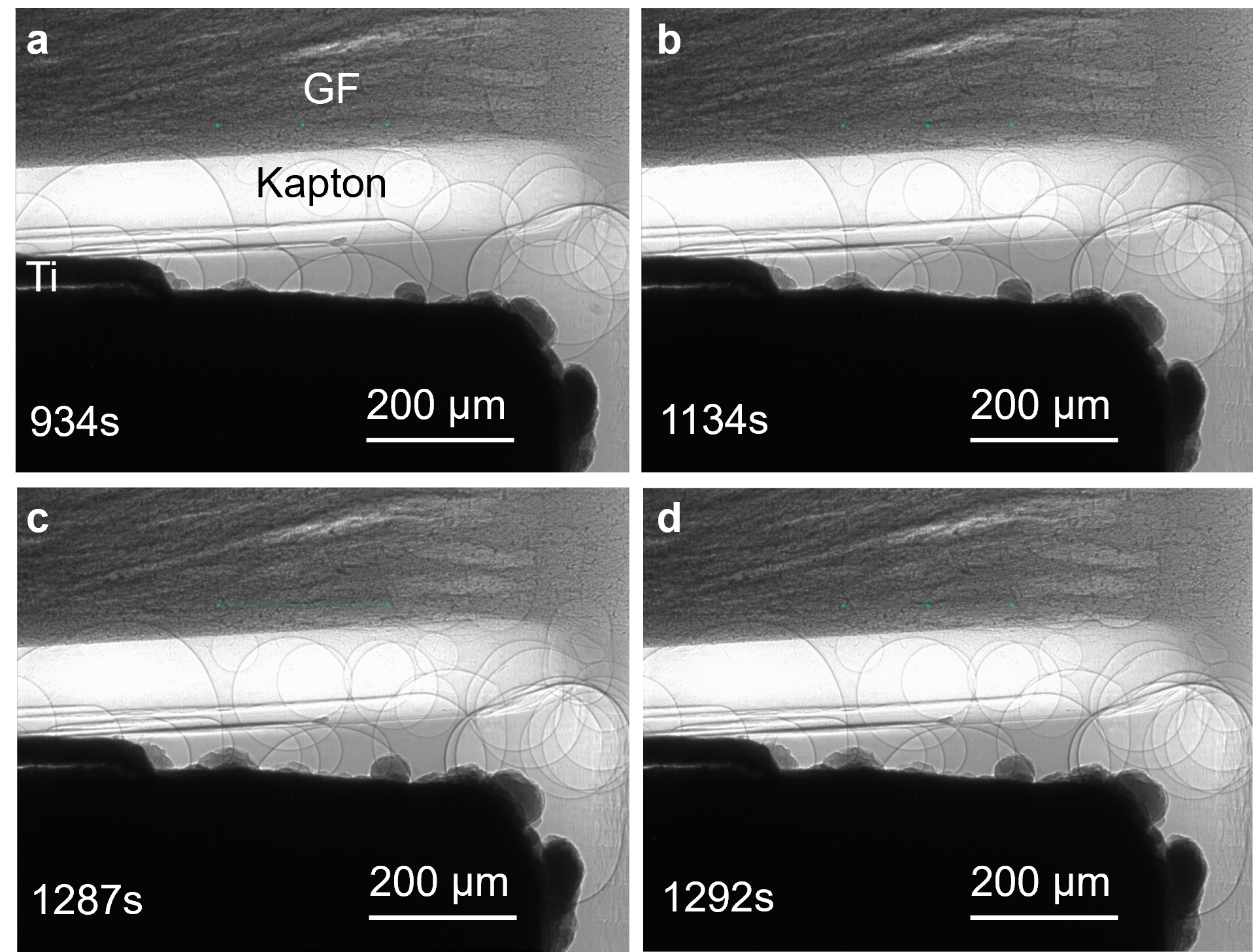


**Fig. S3** Operando X-ray images of the edge of the mounted cells at different deposition states

As shown in Fig. 4 in the main text, the prototype and mounted cells failed much earlier than the designed real-service-inspired cell owing to amplified Zn dendrite formation and HER. Interestingly, mounted cells failed even earlier than the prototype cells, which may originate from the more severe Zn dendrite formation and HER at the edge of the cell.


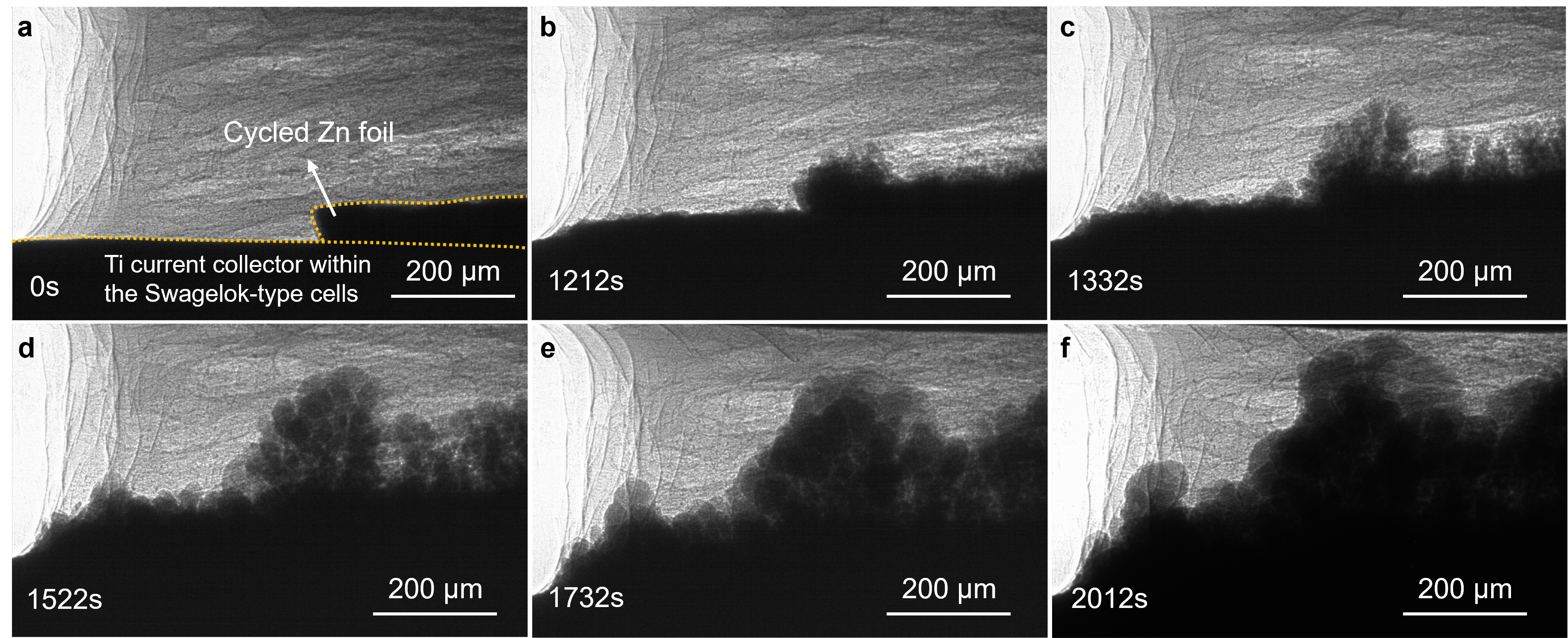


**Fig. S4** Operando X-ray images of designed real-service-inspired cells utilizing cycled Zn foils, which underwent 5 cycles of deposition/stripping at 1 mA cm^-2^


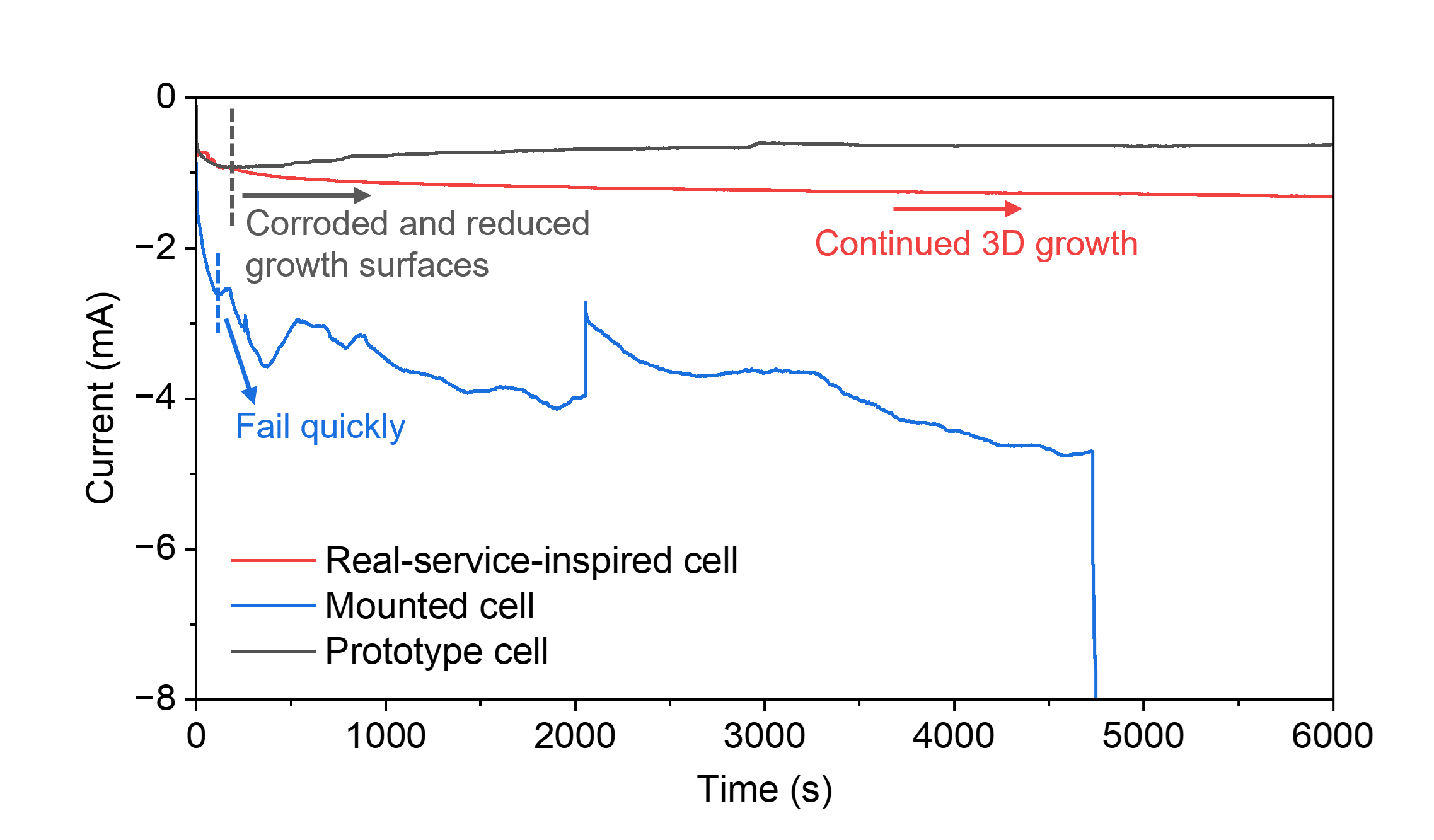


**Fig. S5** Long-term chronoamperograms (CAs) at an overpotential of -150 Mv


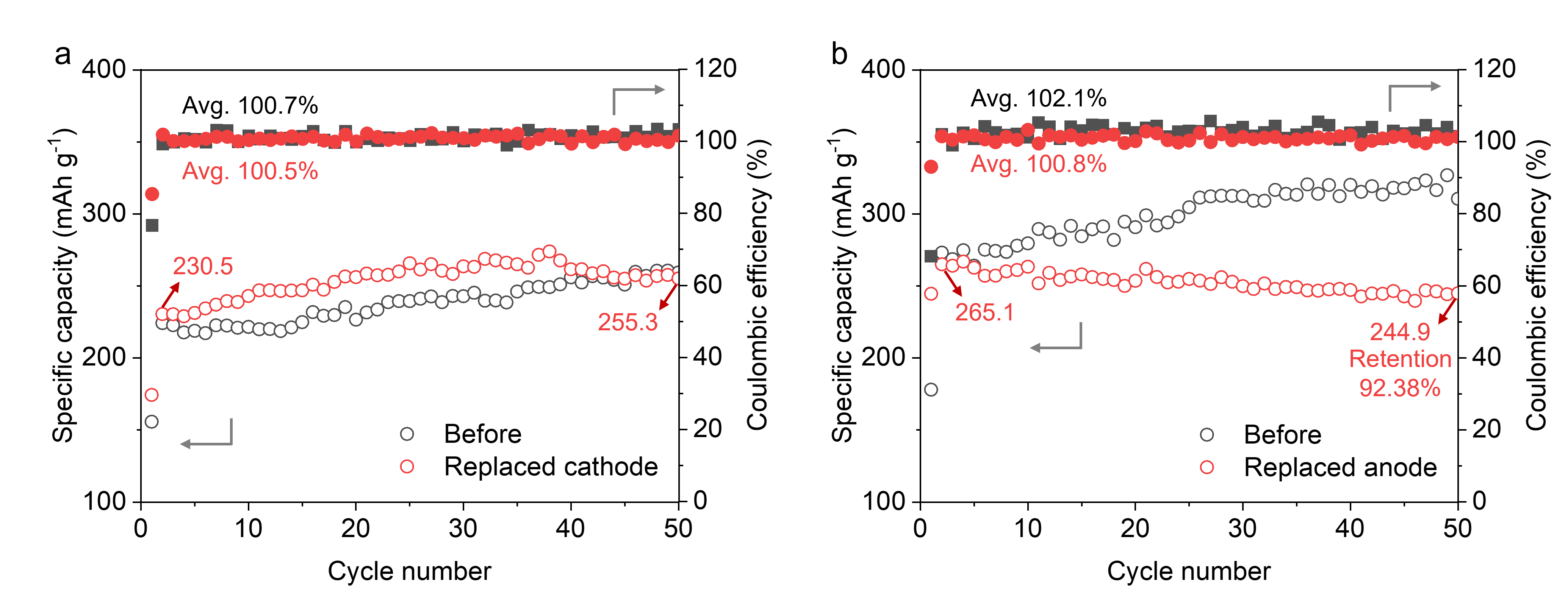


**Fig. S6** Electrode replacement cycling performance of Zn||MnO_2_ coin cells at a current density of 0.5 A g^-1^. (**a**) Cycling performance before and after replacing the MnO_2_ cathode after 50 cycles. (**b**) Cycling performance before and after replacing the Zn foil anode after 50 cycles


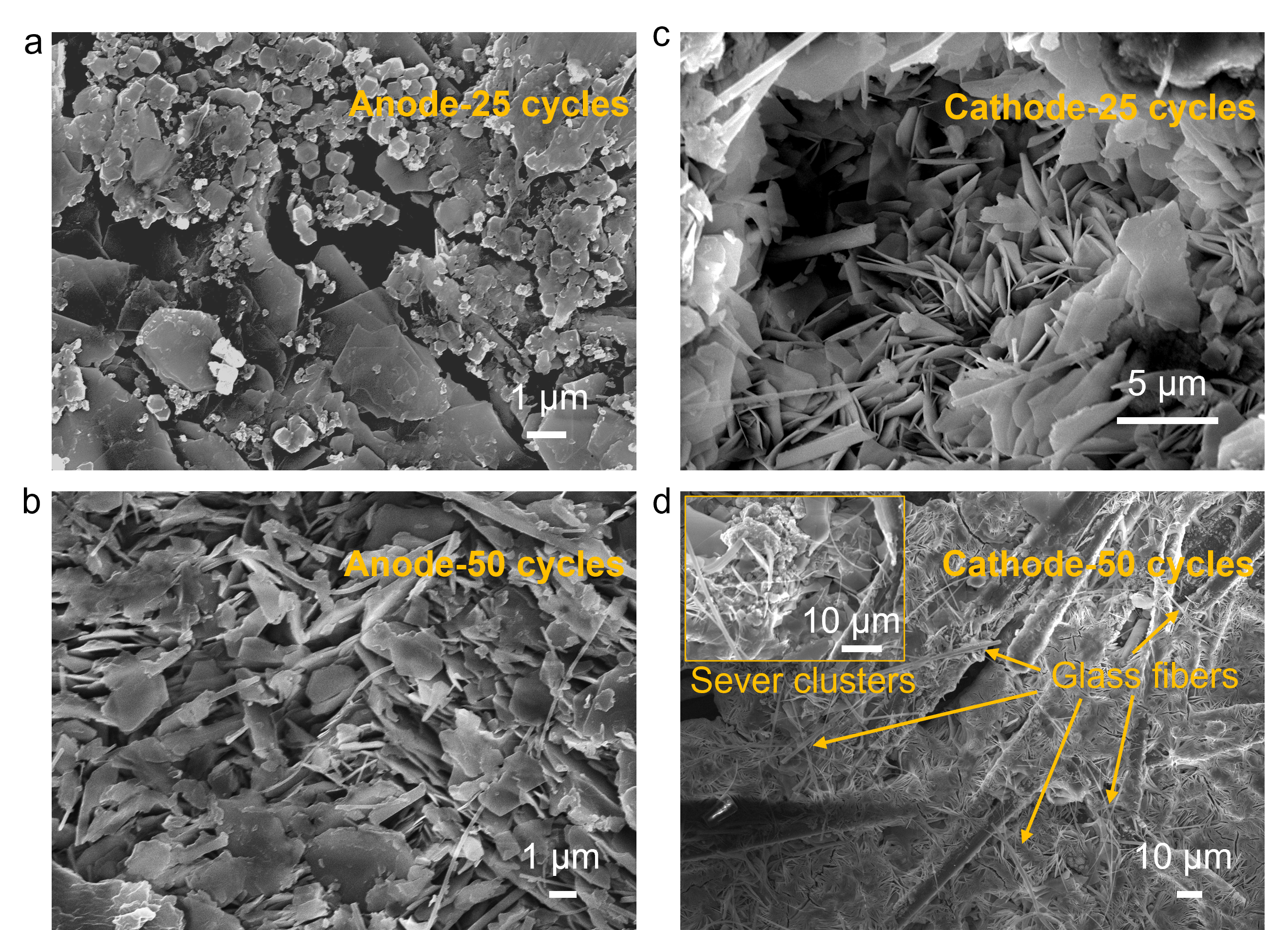


**Fig. S7** SEM images of (**a, b**) Zn foil anodes and (**c, d**) MnO_2_ cathodes disassembled from Zn||MnO_2_ coin cells after 25 cycles and 50 cycles


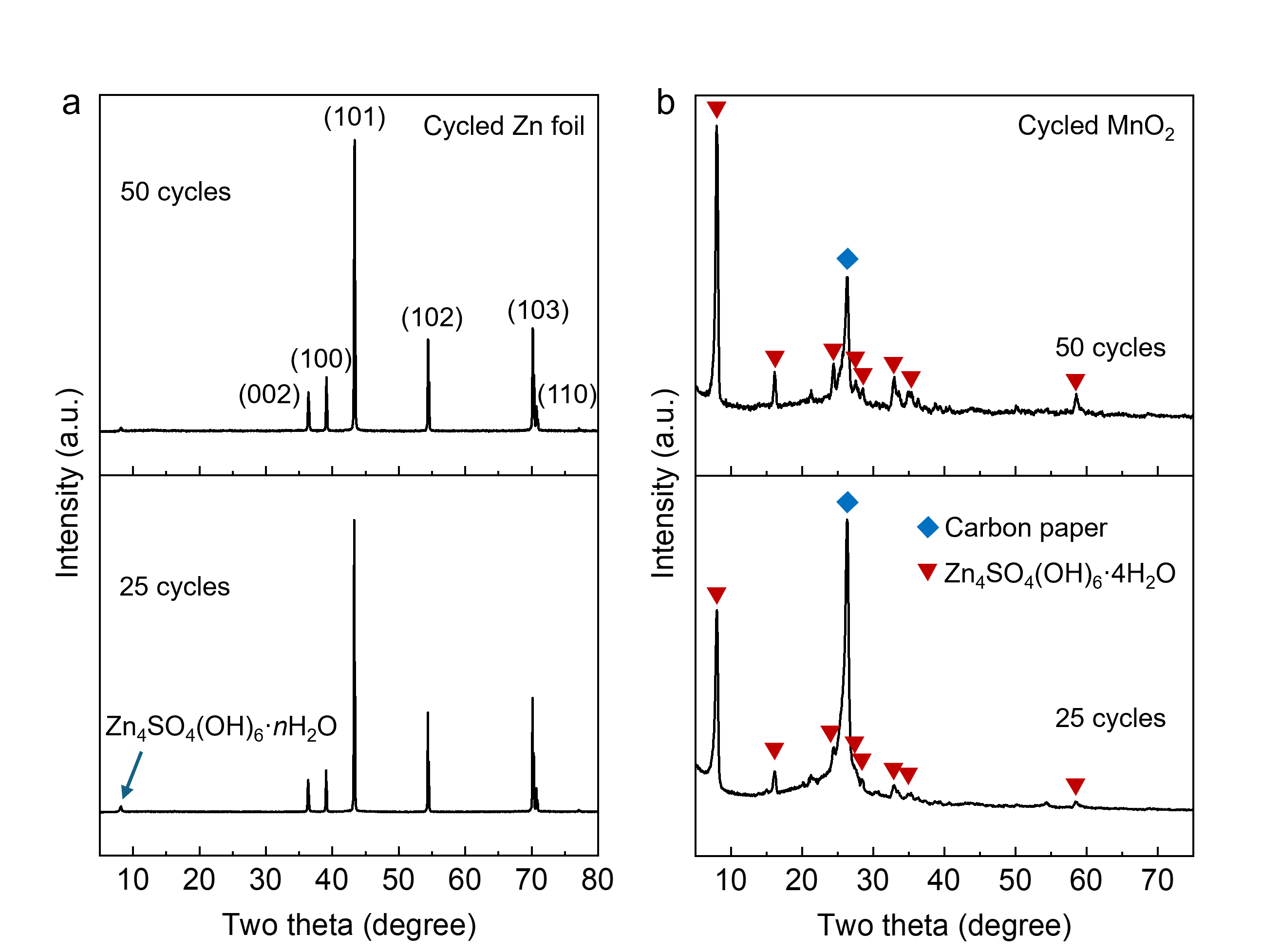


**Fig. S8** XRD patterns of (**a**) Zn foil anodes and (**b**) MnO_2_ cathodes disassembled from Zn||MnO_2_ coin cells after 25 cycles and 50 cycles


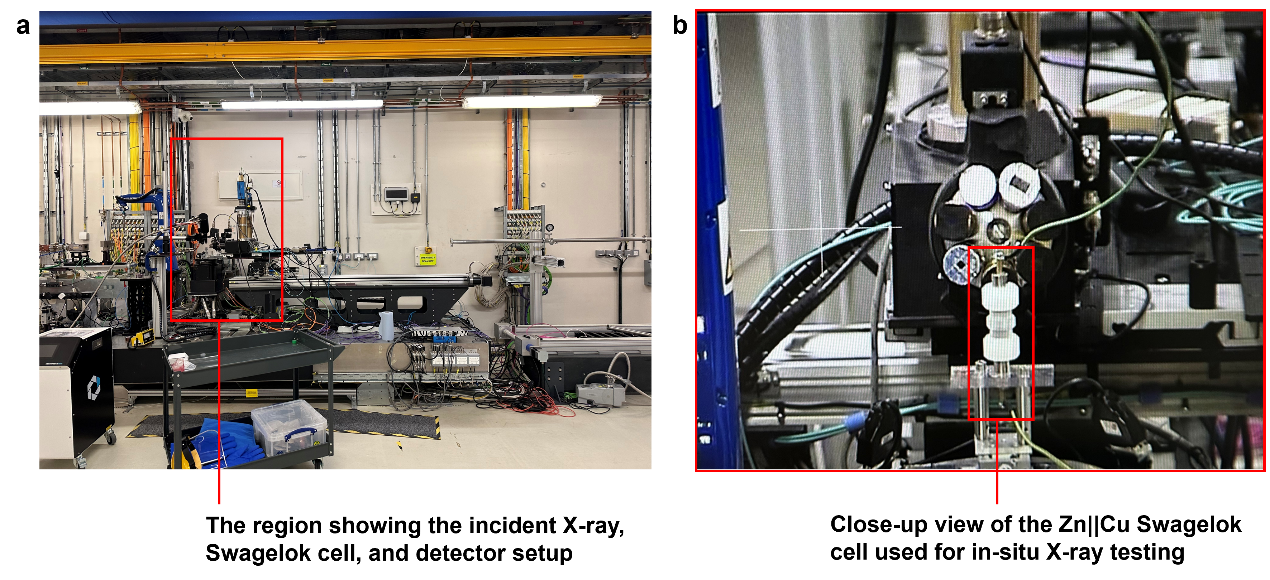


**Fig. S9** Synchrotron X-ray imaging facility at the Diamond Light Source. (**a**) Overall experimental setup showing the X-ray beamline, Swagelok cell, and detector alignment. (**b**) Close-up view of the Zn||Cu Swagelok cell and probe region used for in-situ X-ray imaging

**Supplementary Note**

The parameters of $a$, $b$, $c$, $d$, $I_{0}$, and $\tau_{DL}$ are related to physicochemical parameters as follows:

$$\text{a=}\frac{\text{2·π·n·F·M·h·A·}\text{N}_{\text{0}}\text{·}\text{k}_{\text{1}}^{\text{2}}}{\text{ρ}}\text{ (}\text{S}\text{1)}$$

$$\text{b=}\frac{\text{π·}\text{M}^{\text{2}}\text{·}\text{N}_{\text{0}}\text{·}\text{k}_{\text{1}}^{\text{2}}}{\text{ρ}^{\text{2}}}\text{ (}\text{S}\text{2)}$$

$$\text{c=n·F·A·}\text{k}_{\text{3}}\text{ (}\text{S}\text{3)}$$

$$\text{d=}\frac{\text{π}\text{·M}^{\text{2}}\text{·}\text{N}_{\text{0}}\text{·}\text{k}_{\text{2}}^{\text{2}}}{\text{ρ}^{\text{2}}}\text{ (}\text{S}\text{4)}$$

$$\text{I}_{\text{0}}\text{=}\frac{\text{∆E}}{\text{R}}\text{ (}\text{S}\text{5)}$$

$$\text{τ}_{\text{DL}}\text{=R·C (}\text{S}\text{6)}$$

Symbol meanings are as follows:

$N_{0}$— number density of 2D or 3D active sites formed instantaneously;

$k_{1}$— rate constant of the 2D growth of the nucleus;

$k_{2}$— rate constant of the 3D parallel (lateral) growth;

$k_{3}$— rate constant of the 3D perpendicular (outward) growth;

$A$— electrode surface area active for 2D or 3D nucleation and growth;

$h$— height of the 2D layer;

$\rho$— density of the deposited 2D layer or 3D layer;

$M$— molar mass;

$n$— number of electrons transferred in the deposition reaction;

$\Delta E$— direct current potential step applied to the electrode;

$R$— solution resistance (equivalent series resistance);

$C$— double layer capacitance (equivalent series capacitance);
